# Minimally invasive surgical techniques in vestibular function preservation in patients with cochlear implants

**DOI:** 10.3389/fnins.2022.900879

**Published:** 2022-09-27

**Authors:** Ruijie Wang, Jianfen Luo, Xiuhua Chao, Haibo Wang, Zhaomin Fan, Lei Xu

**Affiliations:** ^1^Department of Otolaryngology-Head and Neck Surgery, Shandong Provincial ENT Hospital, Shandong University, Jinan, China; ^2^Department of Auditory Implantation, Shandong Provincial ENT Hospital, Jinan, China

**Keywords:** cochlear implantation, minimally invasive surgery, vestibular function, otolith, canal

## Abstract

**Background:**

Cochlear implantation (CI) is an effective and successful method of treating individuals with severe-to-profound sensorineural hearing loss (SNHL). Coupled with it’s great clinical effectiveness, there is a risk of vestibular damage. With recent advances in surgical approach, modified electrode arrays and other surgical techniques, the potential of hearing preservation (HP) has emerged, in order to preserve the inner ear function. These techniques may also lead to less vestibular damage. However, a systematic study on this at different follow-ups after CI surgery has not been documented before.

**Aims:**

To investigate changes of vestibular function systematically in recipients at short and long follow-ups after a minimally invasive CI surgery.

**Methods:**

In this retrospective study, 72 patients (72 ears) with minimally invasive CI were recruited. All participants selected had bilateral SNHL and pre-operative residual hearing (RH) and underwent unilateral CI. They were treated to comprehensive care. All patients underwent vestibular function tests 5 days prior to CI. During the post-operative period, follow-up tests were performed at 1, 3, 6, 9, and 12 months. The contemporaneous results of caloric, cervical vestibular-evoked myogenic potential (cVEMP), ocular vestibular-evoked myogenic potential (oVEMP), and video head impulse (vHIT) tests were followed together longitudinally.

**Results:**

On the implanted side, the percent fail rate of caloric test was significantly higher than that of vHIT at 1, 3, and 9 months post-operatively (*p* < 0.05); the percent fail rate of oVEMP was higher than vHIT of superior semicircular canal (SSC), posterior semicircular canal (PSC), or horizontal semicircular canal (HSC) at 1, 3, and 9 months (*p* < 0.05); at 3 and 9 months, the percent fail rate of cVEMP was higher than that of SSC and PSC (*p* < 0.05). There were no significant differences in the percent fail rates among all tests at 6 and 12 months post-CI (*p* > 0.05). The percent fail rates showed decreased trends in caloric (*p* = 0.319) and HSC tested by vHIT (*p* = 0.328) from 1–3 to 6–12 months post-operatively. There was no significant difference in cVEMP between 1–3 and 6–12 months (*p* = 0.597). No significant differences on percent fail rates of cVEMP and oVEMP between short- and long-terms post-CI were found in the same subjects (*p* > 0.05). Before surgery, the abnormal cVEMP and oVEMP response rates were both lower in patients with enlarged vestibular aqueduct (EVA) than patients with a normal cochlea (*p* = 0.001, 0.018, respectively).

**Conclusion:**

The short- and long-term impacts on the vestibular function from minimally invasive CI surgery was explored. Most of the vestibular functions can be preserved with no damage discrepancy among the otolith and three semicircular canal functions at 12 months post-CI. Interestingly, a similar pattern of changes in vestibular function was found during the early and the later stages of recovery after surgery.

## Introduction

Cochlear implantation (CI) has been widely applied in individuals with severe-to-profound sensorineural hearing loss (SNHL). Although CI is an effective and safe procedure, there is risk of trauma to the vestibular sensor, causing vertigo, balance disorder, or complete deterioration (Ibrahim et al., 2017; Yong et al., 2019; Li and Gong, 2020; Wang et al., 2021; West et al., 2021). Possible reasons include injury during electrode insertion, loss of perilymph, labyrinthitis, endolymphatic hydrops, and electrical stimulation (Fina et al., 2003). However, little is known about the main factors influencing status of vestibular function after CI.

Nowadays, individuals with residual hearing (RH) are also candidates for CI. Optimization of the electrode array and surgical techniques has resulted in a more effective approach to cochlear function preservation. A meta-analysis based on hearing preservation (HP) in cochlear implant surgery showed that a combination of round window (RW) approach with implanting a straight electrode might result in HP (Snels et al., 2019). Glucocorticoids have also been used for their anti-inflammatory and anti-apoptotic properties (Douchement et al., 2015). A recent study on minimally invasive surgery suggested that minimizing intracochlear pressure (ICP) during electrode insertion was effective for HP (Ordonez et al., 2019). Patients with pre-operative RH implanted through these techniques can extensively preserve their cochlear function in the long term (Skarzynski et al., 2019; Sprinzl et al., 2020).

Recently, vestibular function preservation using minimally invasive CI surgery has been addressed. We hypothesized that these techniques could similarly preserve vestibular function because of the proximity of the cochlea and vestibule. The notion rests on the assumption that the primary and secondary effect of insertion trauma might influence peripheral vestibular receptors and cochlear function alike (Stuermer et al., 2019). Tsukada and Usami (2021) found that the risk of vestibular damage could be reduced through less traumatic surgical techniques, such as a RW approach and flexible electrodes. Sosna-Duranowska found that the RW approach in HP techniques was associated with vestibular function protection (Sosna-Duranowska et al., 2021). Other studies have demonstrated that vestibular function can be seriously damaged, even with a RW approach (Li and Gong, 2020; Boje Rasmussen et al., 2021). Nevertheless, systematic studies of the influence of minimally invasive surgery on vestibular function protection are rare.

In previous evaluation of vestibular function in patients with CI, there was an exhaustive analysis of the horizontal semicircular canal (HSC) by caloric stimulation evaluating the low frequency response, otolith function evaluated by cervical vestibular-evoked myogenic potential (cVEMP) and ocular vestibular-evoked myogenic potential (oVEMP), and three semicircular canals evaluated using the video head impulse test (vHIT) with a high frequency stimulation.

We sought to assess the changes in both canal and otolith functions in patients undergoing minimally invasive CI techniques at different follow-up times.

## Materials and methods

### Participants

Seventy-two patients (72 ears) with pre-operative low-frequency residual hearing (LFRH) and severe-to-profound SNHL who underwent minimally invasive CI surgery at our auditory implant department between June 2017 and November 2020 were included in this retrospective study. The inclusion criteria was at least one low-frequency pure tone threshold (125, 250, or 500 Hz) ≤85 dB HL before surgery. Patients with severe cochlear malformation, peripheral vestibular disease, auditory synaptopathy/neuropathy, cochlear fibrosis, previous otologic surgeries, and those at risk to show poor participation were excluded, except for those with enlarged vestibular aqueduct (EVA). Computed tomography (CT) of the temporal bone and magnetic resonance imaging (MRI) were performed pre-operatively. An EVA was defined as a vestibular aqueduct diameter of >1.5 mm at the midpoint between the posterior cranial fossa and inner ear vestibule (Valvassori and Clemis, 1978).

Of these patients, 23 were female and 49 were male, and the mean age at implantation was 20.35 ± 19.05 years (range, 3–67 years). Young patients comprised 46 participants <18 years (mean age at implantation: 8.17 ± 3.49 years, 3–17 years), and adults comprised 26 participants ≥18 years (mean age at implantation: 41.88 ± 15.91 years, 19–67 years). Pre-operative CT and MRI revealed bilateral EVA in 33 (45.83%) participants. A total of 33 and 39 recipients underwent implantation in the left and right ears, respectively. The Nucleus CI422, CI522, and CI24RECA electrodes were implanted in 25, 8, and 17 patients, respectively. A MedEL Flex 28 electrode was implanted in 18 patients. Four recipients underwent implantation with a Nurotron CS-10A electrode. The RW surgical procedure was applied to the Nucleus CI422/522, Med-EL FLEX 28, and Nurotron CS-10A electrodes in 55 (76.39%) patients. The extended RW approach was applied to the Nucleus CI24RECA electrode in 17 patients.

Patients underwent vestibular assessments through caloric, cVEMP, oVEMP, and vHIT tests 5 days prior to CI and again at 1, 3, 6, 9, and 12 months post-CI. However, some patients were lost to follow-ups because of the limitations encountered in clinical settings. The processors were all switched off during tests after implantation. Detailed demographic information on the study participants is presented in Tables 1, 2.

**TABLE 1:** Demographic information of all subjects who participated in this study.

S	Sex	Side	AAT (year)	Electrode	Imaging	Post-CI (month)	Pre-CI VF	LFRH pre-CI (dB HL)
S1	M	R	6	CI422	M, E	1, 6, 9, 12	−, +, +, +, +, +	N, 70, 95
S2	M	L	11	CS-10A	M, E	12	+, +, +, +, +, +	N, 75, 80
S3	F	R	5	CI422	M, E	1, 3, 6, 9	+, +, +, +, +, +	N, 75, 95
S4	M	R	13	CI422	Normal	1, 9	/, +, +, +, +, +	65, 75, 85
S5	M	L	13	CI422	Normal	1, 3, 6, 9, 12	+, +, +, +, +, +	10, 20, 75
S6	M	R	7	F28	Normal	9	+, +, +, +, +, +	N, 75, 95
S7	F	L	6	CI522	Normal	3, 6, 9, 12	−, +, +, +, +, +	N, 65, 80
S8	M	L	5	CI422	M, E	1, 6, 12	/, +, +, +, +, +	N, 80, 80
S9	F	R	17	CI422	M, E	1, 3, 9, 12	+, +, +, +, +, +	65, 75, 90
S10	M	R	7	CI422	M, E	1, 3, 6, 12	/, +, +, +, +, +	N, 55, 60
S11	M	R	14	CI422	M, E	1, 3, 9, 12	+, +, +, +, +, +	75, 85, 95
S12	M	R	8	CI422	M, E	6, 9, 12	−, +, +, +, +, +	70, 65, 75
S13	F	R	6	CI422	M, E	6	+, +, +, +, +, +	N, 75, 95
S14	F	R	10	F28	Normal	1, 3, 6, 9, 12	+, +, +, +, +, +	50, 65, 85
S15	M	L	19	F28	M, E	3, 9	+, +, +, +, +, +	65, 80, 85
S16	M	R	12	F28	Normal	1, 6, 9, 12	+, +, −, +, +, +	30, 45, 100
S17	F	L	6	F28	M, E	1, 6, 12	/, +, +, +, +, +	N, 65, 55
S18	M	R	7	CI422	Normal	3, 6, 9, 12	/, +, +, +, +, +	55, 75, 100
S19	M	R	3	CI422	Normal	1, 6, 12	/, −, +, +, +, +	N, 60, 70
S20	F	R	11	F28	M, E	3, 6, 9	+, +, +, +, +, +	60, 70, 80
S21	F	L	35	CI422	Normal	1, 3, 6, 9, 12	+, −, +, +, +, +	55, 50, 50
S22	M	L	62	F28	Normal	1, 3, 9, 12	−, −, −, +, +, +	40, 45, 60
S23	F	R	34	CI522	Normal	1, 3, 6, 9	+, +, +, +, +, +	20, 25, 45
S24	M	R	10	CI422	Normal	3, 6, 12	+, +, +, +, +, +	45, 65, 95
S25	M	L	6	F28	Normal	12	−, +, −, +, +, +	N, 80, 85
S26	M	R	7	CI422	M, E	1, 3, 9	+, +, +, +, +, +	N, 70, 90
S27	F	L	67	CI422	Normal	6, 12	−, −, −, +, +, +	55, 65, 70
S28	F	L	15	F28	M, E	9	−, +, −, +, +, +	60, 70, 75
S29	M	R	6	CI422	M, E	1, 12	/, +, +, +, +, +	55, 45, 70
S30	M	L	6	CI522	M, E	1, 12	/, +, +, +, +, +	60, 45, 65
S31	M	L	48	CS-10A	Normal	1, 3, 6	/, −, −, +, +, +	65, 70, 95
S32	M	L	5	CI422	M, E	3, 6	/, +, +, /, /, /	70, 70, 70
S33	M	L	6	F28	M, E	3	/, +, +, +, +, +	60, 65, 75
S34	M	L	7	CI522	M, E	1, 3	+, +, +, +, +, +	70, 70, 70
S35	M	L	41	CS-10A	Normal	1	+, +, +, +, +, +	50, 70, 100
S36	M	R	52	F28	Normal	1, 3	−, +, −, +, −, −	50, 70, 80
S37	F	L	67	CI422	Normal	1, 6, 12	−, −, −, +, +, +	55, 65, 70
S38	F	R	54	CI522	Normal	1, 3, 12	−, +, +, +, +, +	45, 45, 55
S39	M	L	5	CI422	M, E	1, 3, 9	+, +, +, +, +, +	65, 55, 55
S40	M	R	5	CI522	M, E	1, 3, 9	+, −, −, +, +, +	50, 50, 60
S41	F	R	53	CS-10A	Normal	1, 3, 6	−, −, −, +, −, +	40, 55, 75
S42	M	R	6	F28	M, E	1, 3, 6	+, +, +, +, +, +	80, 75, 90
S43	F	L	11	CI422	Normal	1, 3	/, +, +, +, +, +	60, 75, 90
S44	M	R	5	CI422	M, E	1, 6	−, +, +, +, +, +	N, 60, 85
S45	M	R	34	CI422	Normal	6, 12	+, +, −, +, +, +	N, 85, 90
S46	F	R	7	F28	Normal	1, 3	−, +, +, +, +, +	N, 85, 85
S47	F	L	9	F28	M, E	1, 6	+, +, +, +, +, +	55, 65, 95
S48	F	R	9	F28	M, E	1, 6	+, +, −, +, +, +	60, 70, 90
S49	M	L	11	F28	Normal	3, 9, 12	−, +, +, +, +, +	70, 80, 90
S50	M	L	29	CI422	Normal	1	−, −, +, +, +, +	60, 70, 85
S51	M	L	8	F28	E	1, 3, 9	−, −, −, +, +, −	85, 90, 105
S52	F	R	7	CI422	M, E	1, 3, 6	−, +, +, +, +, −	55, 60, 65
S53	M	L	20	CI522	Normal	1, 9	+, +, +, +, +, +	30, 40, 55
S54	M	R	20	CI522	Normal	1, 9	+, +, +, +, +, +	30, 45, 90
S55	M	R	15	F28	Normal	1, 3	+, +, +, +, +, +	75, 85, 95
S56	M	R	6	CA	M, E	1, 3, 6, 9, 12	+, +, +, +, −, +	N, 65, 75
S57	M	L	8	CA	M, E	1, 3, 9, 12	−, +, +, +, +, +	85, 85, 90
S58	M	R	53	CA	Normal	12	−, −, −, −, −, −	N, 75, 80
S59	F	L	28	CA	Normal	1, 3, 6, 9	−, +, +, +, +, +	15, 45, 80
S60	M	L	19	CA	Normal	1, 3, 6, 9	+, −, /, +, +, +	85, 85, 90
S61	M	R	48	CA	Normal	1, 3	+, −, −, +, −, −	80, 80, 75
S62	F	R	50	CA	Normal	6, 9, 12	−, −, −, +, +, +	65, 75, 85
S63	F	R	6	CA	E	1, 9	+, +, +, +, +, +	N, 80, 90
S64	M	L	30	CA	Normal	1, 3, 9	+, +, +, +, +, +	N, 80, 100
S65	M	R	65	CA	Normal	3	−, −, −, +, +, +	75, 80, 80
S66	M	L	55	CA	Normal	1, 9	+, −, −, +, +, +	N, 80, 65
S67	M	R	6	CA	M, E	3, 9	/, +, +, /, /, /	N, 85, 105
S68	F	L	36	CA	Normal	1, 3	+, +, −, +, +, +	65, 70, 100
S69	M	L	4	CA	E	1, 9	+, +, +, +, +, +	N, 50, 80
S70	M	R	5	CA	M, E	3	−, +, +, +, +, +	N, 80, 95
S71	M	L	51	CA	Normal	1, 3	−, +, +, −, +, +	N, 70, 70
S72	M	R	19	CA	Normal	1, 6	+, −, −, +, +, +	65, 65, 80

AAI, age at implantation; CA, CI24RECA; F, female; M, male; L, left; R, right; M, Mondini; E, enlarged vestibular aqueduct; LFRH, low frequency residual hearing (125, 250, 500 Hz); N, not tested; pre-CI VF, vestibular function pre-operatively in the following order (caloric, cVEMP, oVEMP, SSC, HSC, PSC); /, no tested; +, normal response; −, absent or decreased response; HSC, horizontal semicircular canal; SSC, superior semicircular canal; PSC, posterior semicircular canal.

**TABLE 2:** Summary the number of patients tested at all the time points.

Tests	Number of patients tested among all 72 patients					
	
	Pre	1 Month	3 Months	6 Months	9 Months	12 Months
Caloric	59	28	27	20	21	20
cVEMP	72	43	36	28	30	24
oVEMP	71	43	36	27	29	24
SSC	70	45	40	32	32	28
HSC	70	45	40	32	32	28
PSC	70	45	40	32	32	28

### Minimally invasive surgical techniques

All participants underwent surgery performed by a single surgeon. Full insertion of the electrode was achieved in all patients. In addition to the approach toward the insertion point during surgery and choice of electrode array, other protective measures included the following: (1) the ossicle chain was kept intact during surgery; (2) rotational speed was reduced to a minimum to avoid sound damage when grinding the bone of the RW niche or extending RW; (3) care was taken to avoid aspiration of perilymphatic fluid; (4) sodium hyaluronate was used before opening the RW membrane; (5) the electrode was inserted steadily, gently, and slowly with an insertion time >1 min; (6) after electrode insertion, a small piece of muscle was gently packed around the RW; and (7) systematic glucocorticoids were administered to all patients 1 day before surgery until 1 week after surgery.

### Caloric test

The bithermal caloric test was performed. A video-based system was used (Ulmer VNG, v. 1.4; Synapsys, Marseille, France) to acquire and analyze the eye response. Each ear was irrigated alternatively with a constant flow of air at 24 and 49°C for 40 s. The response was recorded over 3 min. A 7-min interval between each stimulus was observed to avoid cumulative effects. We calculated the maximum slow-phase velocity (SPV) of nystagmus after each irrigation to determine unilateral weakness (UW) according to Jongkee’s formula. In our laboratory, a value of UW less than 20% was judged normal.

### Cervical vestibular-evoked myogenic potential

Cervical vestibular-evoked myogenic potential was recorded using the Neuro-Audio auditory evoked potential equipment (Neurosoft Ltd., Ivanovo, Russia). The test was performed with the patients in seated position. Tone burst stimuli (93–100 dB nHL, 500 Hz) were delivered via a standard headphone. Active recording electrodes with respect to the examination were placed on the region of the upper third of the sternocleidomastoid muscle (SCM) on both sides. The reference electrodes were placed on the upper sternum. The ground electrode was on the nasion. The head was rotated toward the contralateral side of the stimulated ear to achieve tonic contraction of the SCM during recording. The stimulation rate was 5.1 Hz. Bandpass filtering was 30–2000 Hz. An amplitude ratio over 30% was considered abnormal if the weaker response was from the implanted ear. In the event of bilaterally absent responses, the absent response was considered abnormal.

### Ocular vestibular-evoked myogenic potential

Ocular vestibular-evoked myogenic potential was recorded using the Neuro-Audio auditory evoked potential equipment (Neurosoft Ltd., Ivanovo, Russia). The electromyographic activity of the extraocular muscle was recorded with the patients in the seated position. Tone burst stimuli (93–100 dB nHL, 500 Hz) were delivered via a standard headphone. The active recording electrodes were placed on the infra-orbital ridge 1 cm below the center of each lower eyelid. The reference electrodes were positioned approximately 1 cm below them. The ground electrode was on the nasion. The results were recorded with eyes open and maximal gaze upward. The stimulation rate was 5.1 Hz. Bandpass filtering was 1–1000 Hz. An amplitude ratio over 30% was considered abnormal if the weaker response was from the implanted ear. In the event of bilaterally absent responses, the absent response was considered abnormal (Zhang et al., 2019).

### Video head impulse test

The vHIT device (Ulmer II Evolution, France) was used. The patient was instructed to maintain eye fixation on a stationary object on a screen at about 1 m distance while examiner manipulated the patient’s head with quick and precise head movements. The vestibulo-ocular reflex (VOR) gain was calculated by vHIT software based on head velocity and eye velocity curves. In a full test, 5–10 head thrusts were completed per canal for the recording. When the head was turned in the plane of the semicircular canal to be tested, the VOR maintained visual fixation. The breaking of visual fixation, revealed by a corrective saccade, indicated a respective canal disorder. This test was possible as soon as the child could hold his head steady. A VOR gain of the HSC less than 0.8 was considered to be abnormal. For both the superior semicircular canal (SSC) and posterior semicircular canal (PSC), a VOR gain less than 0.7 was considered to be abnormal (Sichnarek et al., 2019).

### Statistical analyses

Data were analyzed using the Statistical Package for the Social Sciences (SPSS), version 23.0 (SPSS, Inc., Chicago, IL, USA). The Chi-square test was used to compare the percent fail rates. Statistical significance was set at *p* < 0.05.

## Results

### Comparison of percent fail rates among each vestibular end-organ senor on the implanted side at different time points

Of the 72 patients, 25 were evaluated with all four assessments before CI and 1 month after CI. Caloric responses in CI ears were normal in 18 cases pre-operatively and abnormal in 44.44% (8/18) of cases 1 month post-operatively. Similarly, the percent fail rates were 27.27% (6/22) for cVEMP, 47.62% (10/21) for oVEMP, 8.33% (2/24) for SSC, 16.00% (4/25) for vHIT of HSC, and 8.33% (2/24) for PSC at 1 month post-operatively. The chi-square test showed that the percent fail rate of caloric was significantly higher than that of SSC, HSC, and PSC tested under vHIT (*p* = 0.019, 0.040, and 0.019, respectively). The rate of oVEMP was higher than that of SSC, HSC, and PSC (*p* = 0.003, 0.020, and 0.003, respectively).

At 3 months post-operatively, the percent fail rate was significantly higher in caloric than in SSC, HSC, and PSC (*p* = 0.002, 0.036, and 0.009, respectively); the rate of oVEMP was higher than that of SSC and PSC (*p* = 0.011, 0.039); and the rate of cVEMP was higher than that of SSC and PSC (*p* = 0.015, 0.049).

At 9 months post-operatively, the percent fail rate was significantly higher in caloric than in SSC, HSC, and PSC (*p* = 0.002, 0.016, and 0.004, respectively); the rate of oVEMP was higher than that of SSC, HSC, and PSC (*p* = 0.004, 0.029, and 0.007, respectively); and the rate of cVEMP was higher than that of SSC and PSC (*p* = 0.020, 0.032).

No statistically significant differences were observed in the percent fail rates among all vestibular function tests at 6 and 12 months after implantation (*p* > 0.05).

**FIGURE 1:**
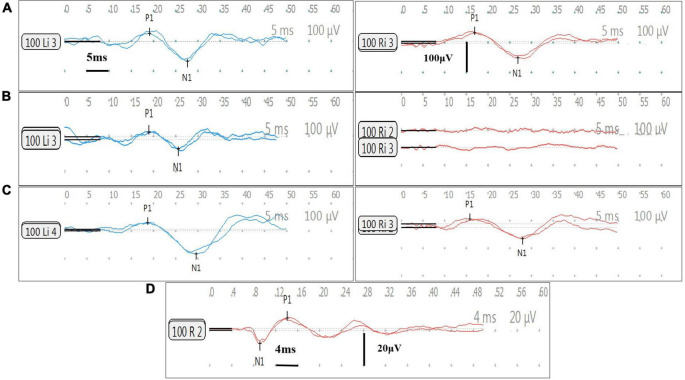
Cervical vestibular-evoked myogenic potential (cVEMP) and oVEMP responses of Subject 9. All results are obtained in Subject 9. Her right side is implanted. The left side is blue and the right side is red. The cVEMP response (positive P1 first) and oVEMP response (negative N1 first) are recorded at 100 dB HL (500 Hz tone burst). In cVEMP, the horizontal and vertical calibrations are 5 ms and 100 μV between two adjacent row of points, respectively. In oVEMP, the horizontal and vertical calibrations are 4 ms and 20 μV, respectively. The two traces show the responses of the repeat stimulus. The amplitude ratio (AR) is defined as the difference between the amplitudes of two sides divided by the sum of the amplitudes of two sides. **(A)** Normal bilateral cVEMP responses before surgery. The amplitude is 92.9 μV on the left side and is 82.8 μV on the right, with a AR of 5.7%. **(B)** Normal left cVEMP response and absent right cVEMP response at 1 month after surgery. The amplitude is 60.5 μV on the left and the AR is 100%. **(C)** Normal bilateral cVEMP responses at 12 months post-surgery. The amplitude is 113.2 μV on the left and is 71.9 μV on the right, with a AR of 22.3%. **(D)** Normal right oVEMP response at 12 months post-surgery. The amplitude is 14.6 μV.

**FIGURE 2:**
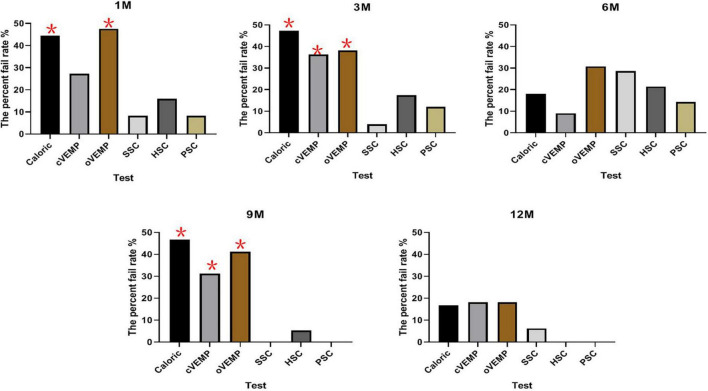
The percent fail rates of all the four tests on the implanted side at each time point. The percent fail rate of caloric was significantly higher than vHIT at 1, 3, and 9 months post-operatively (*p* < 0.05). The percent fail rate of oVEMP was higher than that of SSC, HSC, and PSC at 1 and 9 months; the rate was higher than that of SSC and PSC at 3 months post-operatively (*p* < 0.05). At 3 and 9 months post-operatively, the percent fail rate of cVEMP was higher than that of SSC and PSC (*P < 0.05).

In this part, a different set of patients contributed at each time point for each test. All the post-operative vestibular damages were new damages. cVEMP and oVEMP results are shown in Figure 1. The percent fail rates of all the four tests on the implanted side at each time point are listed in Table 3 and Figure 2.

**TABLE 3:** The simultaneous comparison of percent fail rates of vestibular function on the implanted side at all time points.

Tests	Number of patients tested, Percent fail rate (N,%)				
	
	1 Month *N* = 25	3 Months *N* = 25	6 Months *N* = 15	9 Months *N* = 20	12 Months *N* = 16
Caloric	8/18, 44.44*	9/19, 47.37*	2/11, 18.18	7/15, 46.67*	2/12, 16.67
cVEMP	6/22, 27.27	8/22, 36.36*	1/11, 9.00	5/16, 31.25*	2/11, 18.18
oVEMP	10/21, 47.62*	8/21, 38.10*	4/13, 30.77	7/17, 41.18*	2/11, 18.18
SSC	2/24, 8.33	1/25, 4.00	4/14, 28.57	0/22, 0.00	1/16, 6.25
HSC	4/25, 16.00	4/23, 17.39	3/14, 21.43	1/19, 5.26	0/16, 0.00
PSC	2/24, 8.33	3/25, 12.00	2/14, 14.29	0/19, 0.00	0/16, 0.00

*p < 0.05.

### Changes in percent fail rate in each vestibular end-organ function on the implanted side at short (1–3 months) and long (6–12 months) follow-up times after surgery

To study the same set of patients longitudinally we divided the follow-up time points into two groups: early (1–3 months) and late (6–12 months). Nineteen patients in caloric, 35 in cVEMP, 35 in oVEMP, and 38 in vHIT were evaluated before CI, 1–3 months and 6–12 months post-CI. All of these patients had pre-operative normal vestibular functions. In this part, for each test the same group of patients participated at all three time points.

For a patient who had multiple assessments at different evaluation time points during his follow-up, the first assessment from 1 to 3 months was chosen as the short-term result and the latest assessment from 6 to 12 months was chosen as the long-term result.

In the caloric test, the percent fail rates were 47.37% (9/19) and 31.58% (6/19) at 1–3 (1.57 ± 0.90) months and 6–12 (9.63 ± 2.36) months after surgery, respectively. In cVEMP, the rates were 31.43% (11/35) at 1–3 (1.46 ± 0.85) months and were 25.71% (9/35) at 6–12 (9.69 ± 2.31) months. In vHIT of HSC, the rates were 18.42% (7/38) at 1–3 (1.47 ± 0.86) months and were 10.53% (4/38) at 6–12 (9.79 ± 2.28) months.

**FIGURE 3:**
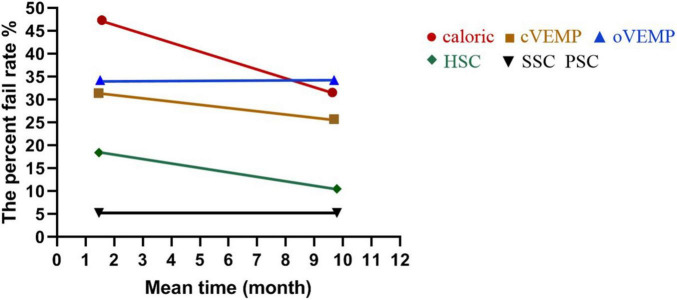
The percent fail rate of each end-organ function on the implanted side at short and long follow-up times after surgery. The percent fail rates showed no significant differences from 1–3 (1.57 ± 0.90) to 6–12 (9.63 ± 2.36) months in caloric (*p* = 0.319) and from 1–3 (1.47 ± 0.86) to 6–12 (9.79 ± 2.28) months in HSC tested by vHIT (*p* = 0.328) post-operatively. There was no significant difference in cVEMP between 1–3 (1.46 ± 0.85) and 6–12 (9.69 ± 2.31) months (*p* = 0.597). The percent fail rates of oVEMP were the same at 1–3 (1.51 ± 0.89) and 6–12 (9.69 ± 2.31) months (*p* = 1.000). The percent fail rates of SSC and PSC were the same at 1–3 (1.47 ± 0.86) and 6–12 (9.79 ± 2.28) months (*p* = 1.000, respectively).

The percent fail rates showed decreased trends from 1–3 to 6–12 months in caloric (*p* = 0.319) and HSC tested by vHIT (*p* = 0.328), but the trend did not reach statistical significance. There was no significant difference in cVEMP between 1–3 and 6–12 months after surgery (*p* = 0.597). The percent fail rates of oVEMP were the same at 1–3 (1.51 ± 0.89) months and at 6–12 (9.69 ± 2.31) months post-operatively (*p* = 1.000). The percent fail rates of SSC and PSC were the same at 1–3 (1.47 ± 0.86) months and at 6–12 (9.79 ± 2.28) months (*p* = 1.000, respectively). The percent fail rates of each end-organ function on the implanted side at short and long follow-up times after surgery are shown in Table 4 and Figure 3.

**TABLE 4:** The percent fail rate of each end-organ function on the implanted side at all five time points after surgery.

Tests (total patient number)	Number of patients with percent fail rates (*N*, %)	
	
	1–3 Months	6–12 Months
Caloric (19)	9, 47.37	6, 31.58
cVEMP (35)	11, 31.43	9, 25.71
oVEMP (35)	12, 34.29	12, 34.29
SSC (38)	2, 5.26	2, 5.26
HSC (38)	7, 18.42	4, 10.53
PSC (38)	2, 5.26	2, 5.26

### Comparison of otolith function variations in the same subjects at short and long follow-up times after surgery

Among these 72 participants, 31 with pre-operative normal otolith functions underwent cVEMP and oVEMP before CI, 1–3 and 6–12 months post-CI simultaneously.

At 1–3 (1.58 ± 0.92) months after surgery, the percent fail rate of cVEMP was 32.26% (10/31), with five children showing decreased responses and five showing absent responses; the percent fail rate of oVEMP was 35.48% (11/31), with three children having decreased responses and eight having absent responses.

At 6–12 (9.87 ± 2.22) months after surgery, the percent fail rate of cVEMP was 25.81% (8/31), with two children showing decreased responses and six children showing absent responses; the percent fail rate of oVEMP was 35.48% (11/31), with two children having decreased responses and nine having absent responses.

There were no significant differences on percent fail rates of cVEMP and oVEMP between short- and long-terms post-CI (*p* > 0.05).

### Comparison of vestibular function between patients with enlarged vestibular aqueduct and patients with a normal cochlea before surgery

Twenty-five patients with EVA and 33 with a normal cochlea underwent all the four tests before surgery. Comparing patients with EVA to normal patients the caloric test showed abnormal responses 32% (8/25) vs. 48.48% (16/33) of the time, respectively; cVEMP 8% (2/25) vs. 36.36% (12/33) of the time, oVEMP 16.00% (4/25) vs. 45.45% (15/33) of the time, vHIT of HSC 4.00% (1/25) vs. 12.12% (4/33) of the time, vHIT of SSC 0.00% (0/25) vs. 3.03% (1/33) of the time, vHIT of PSC 8.00% (2/25) vs. 9.09% (3/33) of the time.

Both the abnormal cVEMP and oVEMP response rates were lower in patients with EVA than patients with a normal cochlea (*p* = 0.001, 0.018, respectively). There were no significant differences in the abnormal response rates between the two patient groups for caloric test, vHIT of SSC, HSC, and PSC (*p* = 0.207, 1.000, 0.536, 1.000, respectively).

## Discussion

In this report, all 72 patients underwent minimally invasive surgical techniques combining the surgical approach, electrode array, slow insertion of electrode, and systematic glucocorticoids. It is known that this surgical procedure plays an important role in protecting the delicate intracochlear structures (Fina et al., 2003; Eshraghi and Van De Water, 2006; Causon et al., 2015; Kuang et al., 2015; Bruce and Todt, 2018). The concepts of atraumatic electrode insertion include implantation through the RW or extension of the RW (Skarzynski et al., 2002). The RW approach with a straight electrode yielded HP result (Snels et al., 2019). Fifty-one (70.83%) of our patients used flexible electrodes. Although 23.61% of our patients used counter electrodes and four recipients implanted with the Nurotron CS-10A electrode, other protective techniques were used. Only one or two evaluation time points were analyzed in a few previous studies on vestibular function protection with soft surgery (Guan et al., 2021; Sosna-Duranowska et al., 2021; Tsukada and Usami, 2021). The variation in vestibular function at different follow-ups during the first year was analyzed in this study for the first time. Therefore, the trajectory of function variation can be followed dynamically and continuously.

The functional discrepancies in this present study were disparate when all five vestibular end sensor functions were compared simultaneously. Although the subjects’ biases were inevitable among different intervals, the results had definite meanings. In this report, at 1 and 3 months post-CI, the percent fail rates of cVEMP and oVEMP were higher than those of a recent report that showed that 19.2% of patients in cVEMP and 17.4% in oVEMP had post-operative function loss at 1–3 months after HP surgery (Sosna-Duranowska et al., 2021). Regarding the vHIT results 4–6 months post-operatively, a similar difference was observed. The main reason for these differences could be our stricter criteria used to judge abnormal responses. In this study, at 6 months after surgery, the percent fail rates of cVEMP and oVEMP were 9.00 and 30.77%, respectively, and were both 18.18% at 12 months. These results were consistent with previous results at 6–12 months after less traumatic CI surgery (Tsukada and Usami, 2021).

Our results revealed that otolith and low-frequency HSC functions were damaged more seriously than high-frequency canal functions in the short-term post-operatively (1 and 3 months). These short-term outcomes agree with the changes affected by conventional surgery, which showed that the otolith sensors were more damaged than three semicircular canal functions and the canal functions were seldom impaired (Ibrahim et al., 2017; Dagkiran et al., 2019; Yong et al., 2019). No selective impairments were found at 6 and 12 months although they existed in the 9th month, indicating that the impairment discrepancies among all sensors began to decrease at nearly 6 months when the distant or secondary effects come into play. Fluctuating hearing changes have been reported in patients after HP surgery. The foreign body response and intracochlear fibro-osseous reaction may contribute to this fluctuation (Foggia et al., 2019; Snels et al., 2019). The reason for our variation in the 9th month may be the individual disparity or other reasons such as foreign body response or fibro-osseous. However, the exact mechanism is unknown. This study discovered no damage discrepancy at 12 months, in contrast with previous reports with the same duration after conventional implantation (Ibrahim et al., 2017; Yong et al., 2019). Our study demonstrated that the atraumatic techniques could diminish the functional impairment at least 1 year post-surgery, being more obvious in the long-term period. It was meaningful and comprehensive to assess function status at multiple time points to display the continuously functional variations.

To deeply explore the status of each function, we evaluated it within the same patient group for each vestibular sensor. Our otolith function damage was less than most of the results through conventional surgery (Verbecque et al., 2017; Wang et al., 2022) and our canal functions were seldom damaged. These results verified the validity of protective surgical techniques. In addition, our results showed a similar status of all the functional variations from 1–3 to 6–12 months although there were decreased tendencies of damage in HSC and saccular functions. Finally, we analyzed the otolith function variation on the same cohort subjects and found the same results. A recent study revealed that conventional surgery could injure all five vestibular end-organ functions and the damage of some functions increased with time (Kwok et al., 2022). Conversely, a different tendency was observed in this report.

With regard to our injury trends, we speculated that the instant mechanical injury produced by electrode insertion might not be the primary damage because the electrode does not come in direct contact with the vestibular organs, although the instant damage could be diminished through our protective methods. Besides this, the secondary or distant effects of surgery may threaten vestibular function, such as inflammation, fibrous tissue formation, or ossification (Fayad et al., 2009). Cochlear fibrosis and new bones can be induced by a traumatic electrode insertion (Fayad et al., 2009; Jia et al., 2016). The acute inflammatory response is due to electrode insertion. Then a chronic phase replaces it because of the foreign body reaction involving macrophages, their derivatives, and lymphocytes (Seyyedi and Nadol, 2014). These influencing factors may mainly participate in vestibular damage (Stuermer et al., 2019). Our surgical methods are believed to diminish the damage from inflammation, fibrous tissue information, or ossification to avoid producing an increased damage with time.

Thirty-three (45.83%) participants revealed an EVA and their abnormal otolith function was less common than patients with a normal cochlea before surgery in this study, consistent with our previous results (Wang et al., 2021). It is hypothesized that the presence of a third window might allow for the activation of VEMP, making the otolith organs more excitable and sensitive to sound stimulation (Govender et al., 2016; Wang et al., 2021). However, the influence of the cochlear anatomical malformation on our results were not analyzed because of the inconsistencies in patients pools at different follow-ups. We mainly focused on the overall effect of protective surgical techniques on vestibular function variations and will explore the influence factors in the future.

## Limitations

The main limitation was that some patients were lost to follow-ups after implantation in the clinic. The number of patients evaluated were disparate in the first part of our results. In the next step, we will evaluate vestibular function variations at five continuous follow-up times in the same cohort of subjects for each vestibular sensor.

## Conclusion

In this study, the variation of vestibular function in the short and long terms after a minimally invasive CI surgery during a 12 months period was explored. Most of the vestibular functions can be preserved with no damage discrepancy among the otolith and three semicircular canal functions at 12 months post-CI. Interestingly, a similar pattern of changes in vestibular function was found during the early and the later stages of recovery after surgery. Both the instant influence of electrode insertion and the indirect or secondary factors show a similar trend on the functional variation of each vestibular end sensor after a minimally invasive CI surgery.

## Data availability statement

The original contributions presented in this study are included in the article/supplementary material, further inquiries can be directed to the corresponding author.

## Ethics statement

The studies involving human participants were reviewed and approved by Medical Ethics Committee of Shandong Provincial ENT Hospital. Written informed consent to participate in this study was provided by the participants’ legal guardian/next of kin.

## Author contributions

LX, HW, RW, and XC contributed to the conception of the work. JL and ZF contributed to the experimental design. RW selected data and performed the analysis. All authors contributed to the interpretation of the data and were involved in writing the manuscript.
